# Adherence to CRC Screening and Surveillance Guidelines when Using Split-Dose Bowel Preparation

**DOI:** 10.1155/2018/8237824

**Published:** 2018-07-02

**Authors:** Stacy B. Menees, H. Myra Kim, Grace H. Elta, Sheryl Korsnes, Philip Schoenfeld

**Affiliations:** ^1^Division of Gastroenterology, University of Michigan Health System, Ann Arbor, MI, USA; ^2^Division of Gastroenterology, Ann Arbor Veterans' Administration Health Care System, Ann Arbor, MI, USA; ^3^John D. Dingell VA Medical Center, Detroit, MI, USA

## Abstract

**Goal:**

To prospectively assess physician recommendations for repeat colonoscopy in an average-risk screening cohort.

**Background:**

Endoscopists' adherence to colorectal cancer screening and surveillance guidelines for repeat colonoscopy have not been well characterized. Furthermore, little is known about patient and colonoscopy factors that are associated with endoscopists' nonadherence to guideline recommendation.

**Study:**

This is a prospective cohort of average-risk patients undergoing colonoscopy for colorectal cancer screening between August 2011 and January 2013. The primary outcome was assessment of physician recommendations for repeat colonoscopy.

**Results:**

462 participants were prospectively enrolled. 13.6% (62) had guideline-inconsistent recommendations. 89% of the guideline-inconsistent recommendations were for an earlier interval. Endoscopists' reports cited suboptimal bowel preparation as the most common reason for earlier repeat colonoscopy. On multivariable analysis, patient split-dose preparation noncompliance was significantly associated with guideline-inconsistent recommendation (OR = 2.7) even after adjusting for other patient or bowel preparation-related characteristics. Additionally, increased odds of guideline-inconsistent recommendation were associated with older age (>70 years old), higher BMI, having 3 or more polyps, having had at least two previous colonoscopies, suboptimal bowel preparation, and having taken at least 12 hours till clear bowel movement.

**Conclusions:**

Gastroenterologists are adherent to CRC screening and surveillance guidelines. Suboptimal bowel preparation is the most frequently cited factor in endoscopy reports leading to deviation from guidelines. Continued emphasis on optimization of bowel preparation, particularly patient compliance to split-dose regimen, is needed.

## 1. Introduction

Colonoscopy is an effective colorectal cancer (CRC) screening test, but it may be overused [1, 2]. This overuse is partly defined as recommending a repeat colonoscopy sooner than 10 years after a normal screening colonoscopy in an average-risk individual aged ≥50 or recommending repeat colonoscopy sooner than 5 years after finding 1-2 nonadvanced adenomas in an average-risk individual [3]. Minimizing overuse of colonoscopy for CRC screening and colon polyp surveillance is a worthy goal because it minimizes patients' exposure to the risks, inconvenience, and cost of colonoscopy. Also, the importance of making guideline-consistent recommendations will only increase. First, the 2015 multisociety position statement on quality indicators in colonoscopy [4] now recommends that guideline-consistent recommendations should be made in at least 90% of cases. Second, recommendation for timing of repeat colonoscopy in an average-risk patient after a normal CRC screening colonoscopy or after finding 1-2 nonadvanced adenomas is an approved quality indicator for the Physician Quality Reporting System (PQRS) instituted by the Centers for Medicare and Medicaid Services (CMS). Third, this quality indicator is also likely to be incorporated into a value-based index of colonoscopy quality by 2017, which will further impact reimbursement for colonoscopy by CMS.

Suboptimal bowel preparation is frequently associated with overuse [5, 6]. Our retrospective review of 1387 average-risk individuals aged ≥50 years with a normal screening colonoscopy found that 24% received recommendations for repeat colonoscopy that were inconsistent with guideline recommendations, and these guideline-inconsistent recommendations were always to have a repeat colonoscopy sooner than recommended by guidelines [5]. We also found that “fair” bowel preparation, as defined by the Aronchick scale, was highly associated with guideline-inconsistent recommendations compared to “excellent” bowel preparation of patients (OR = 18; 95% CI: 12–28). Specifically, only 15% of patients with “excellent” preps received guideline-inconsistent recommendations while 75% of patients with “fair” preps received this type of recommendation. These findings were supported by our second retrospective database study of over 16,000 patients which found that repeat colonoscopy within 5 years was recommended for 70% of average-risk patients with a normal screening colonoscopy and a “fair” prep [7]. This recommendation is understandable since our study also found an adenoma miss rate of 28% when these patients had repeat colonoscopy within 3 years.

Our previously reported data was performed in cohorts that used PM-only bowel preparation as opposed to split-dose bowel preparation, which is superior for bowel cleansing [8–13]. Institution of split-dose bowel preparation could minimize guideline-inconsistent recommendations for timing of repeat colonoscopy by increasing the frequency of optimal bowel cleansing. However, other factors associated with guideline-inconsistent recommendations should be identified in the split-dose bowel preparation era in order to facilitate quality improvement. Therefore, the aim of our study is to assess these issues in a prospective study. With this information, future research can assess interventions to minimize the frequency of guideline-inconsistent recommendations.

## 2. Materials and Methods

### 2.1. Study Design

Eligible patients were average-risk 50–74-year-old individuals undergoing colonoscopy for CRC screening at an ambulatory endoscopy center or a hospital-based endoscopy unit. Exclusion criteria included patients unable to read, comprehend, or consent for their involvement in the study; patients undergoing colonoscopy for abdominal pain, hematochezia, change in bowel habits, or other gastrointestinal symptoms; and patients with a family history of CRC in first-degree relative or previous history of polyps or colon cancer.

Split-dose bowel preparation for all laxative regimens in all patients was instituted uniformly at the study sites in 2010 [14]. At our institution, the standard tool for assessing the quality of bowel preparation is the Aronchick scale (excellent, good, fair, and poor) [15]. For this prospective trial, endoscopists also graded bowel preparation with the Boston bowel preparation scale (BBPS), a valid and reliable instrument [16, 17]. BPPS score can range from 0–9. In BBPS, cleanliness of the right colon, transverse colon, and left colon is assessed separately on a 0–3 scale. BBPS scores were collected after washing and suctioning of residual stool per usual protocol. Colon preparation quality was considered adequate if BBPS was ≥6 [18]. All endoscopists were trained in the BBPS through a didactic session at a mandatory faculty meeting utilizing an instructional video [19]. Additionally, posters of the BBPS scale were posted in every endoscopy room. Endoscopy nurses and study personnel insured proper completion of the BPPS.

After obtaining informed consent from eligible patients before colonoscopy, the following data was collected: age, race/ethnicity, sex, body mass index (BMI), concurrent medical illnesses including presence/absence of diabetes, concurrent medication use including narcotics and tricyclic antidepressant (TCA) usage, marital status, education, employment, income, use of Medicaid insurance, type of bowel preparation agent used, time of colonoscopy, time of first bowel movement after starting bowel preparation, time to first clear bowel movement after starting the bowel preparation (options included: less than 4 hours, between 4–8 hrs, between 9–12 hrs, more than 12 hrs, and never had clear bowel movement), time that bowel preparation was started, and completed for split-dose bowel regimen. Split-dose bowel regimen compliance was assessed with the following survey question: “Please pick the sentence below that best describes how you took the liquid/pill laxative prep.” The participants were given the following choices: (a) “I took all of the liquid/pill laxative prep yesterday”; (b) “I took some of the liquid/pill laxative prep yesterday and some of it today”; (c) “I took all of the liquid/pill laxative prep today”; or (d) “I never took my liquid/pill laxative prep.” Participants who chose “I took some of the liquid/pill laxative prep yesterday and some of it today” were considered compliant with the split-dose bowel regimen.

Colonoscopy data about GI fellow participation, endoscopists' categorization of procedural difficulty, time of day when colonoscopy was performed, number of previous screening colonoscopies performed on the patient, location of procedure (ambulatory endoscopy center versus academic hospital endoscopy unit), number/location/size of polyps, and pathology reports was also collected.

This prospective study was approved by the Institutional Review Board of the University of Michigan.

### 2.2. Endoscopists' Recommendation Intervals

Endoscopists' recommendation for follow-up screening colonoscopy was abstracted from patient colonoscopy reports and follow-up pathology letters. The IRB did not permit the collection of data about specific endoscopists' characteristics so endoscopists could not be singled out, such as by sex or endoscopy site. Follow-up recommendations were labeled as either consistent or inconsistent with guidelines (Table 1). At our institution, if a patient had poor bowel preparation by the Aronchick scale, then the standard recommendation is to repeat colonoscopy within 12 months. This is consistent with the 2012 multisociety guidelines [3]. Failure to provide a recommendation for repeat screening colonoscopy was also considered inconsistent with guideline recommendations.

### 2.3. Statistical Analysis

Recommended follow-up interval colonoscopy was recorded as a dichotomous variable defined as consistent with versus inconsistent with guideline recommendations. Chi-square tests and student *t*-tests were used to assess study population differences in sociodemographic characteristics, bowel preparation quality-related variables, and whether the recommendation was consistent with guidelines. Logistic regression modeling was used to determine independent predictors of follow-up recommendations inconsistent with guidelines. The models were adjusted for bowel preparation type, number of polyps, compliance with split-dose bowel preparation, endoscopy site, sex, race, and concurrent medical illnesses and medications. All models were adjusted for sex and race regardless of statistical significance. Adjusted odds ratios and 95% confidence limits were derived from the final model estimates.

In order to assess for an association between inadequate/poor bowel preparation versus suboptimal bowel preparation versus optimal bowel preparation, the BBPS score was categorized into BBPS 0–4, BBPS = 5, and BBPS = 6–9. Similar analyses were performed for inadequate bowel preparation for a single segment of colon (BBPS = 0-1) or adequate (BBPS 2-3). Also, the Aronchick scale was used to compare poor bowel preparation versus fair bowel preparation versus good/excellent bowel preparation. Database management and statistical analyses were performed using Stata 12.0 (StataCorp LP, College Station, TX).

## 3. Results

Four hundred and sixty-two individuals meeting eligibility were prospectively enrolled. Complete data about bowel preparation quality based on BBPS was missing in six subjects, leaving 456 individuals for inclusion. Mean age of participants was 56.8 years (SD = 6.9), and 50% was male (Table 2). Based on the Aronchick scale, 79% (362/456) had good/excellent bowel cleansing, 14% (63/456) had fair bowel cleansing, 5% (21/456) had poor bowel cleansing, and 2% (10/456) had missing data (Table 3). Based on the BBPS scale, 93.4% (426/526) had an adequate bowel preparation with BBPS ≥ 6 with 2.1% (10/456) having BBPS = 5 and 4.3% (20/456) having BBPS < 5.

Among the 456 eligible patients, 13.6% (62/456) had guideline-inconsistent recommendations. The majority (89%, *N* = 55/62) instructed the patient to get repeat colonoscopy earlier than recommended by guidelines with 6% advising patients to get repeat colonoscopy longer than recommended intervals, and 5% (3/62) of patients did not get any specific recommendation. In bivariate analysis of colonoscopy-associated factors (Table 3), “fair” bowel preparation by the Aronchick scale (*p* < 0.001), BBPS of 0-1 for any colon segment (*p* < 0.001), BBPS total score of 5 (*p* < 0.001), noncompliance with split-dose preparation (*p* = 0.006), and presence of 3 or more polyps (*p* = 0.004), having had at least two prior colonoscopies (<0.001), and having >12 hours or never between starting bowel preparation and first clear bowel movement (*p* = 0.04), were each associated with guideline-inconsistent recommendations (Table 3). In bivariate analyses of patient-associated factors, higher body mass index, ≤high school education, and Medicare eligible status were each associated with guideline-inconsistent physician recommendations (Table 3).

In adjusted multivariable logistic regression analysis, two factors that may be amenable to intervention were independently associated with guideline-inconsistent recommendations: noncompliance with split-dose preparation (OR = 3.6; 95% CI: 1.7–7.7) and having required >12 hours or never till clear stool (OR = 3.4; 95% CI: 1.4–6.6) (Table 4). In addition, multiple patient-related characteristics not amenable to intervention were associated with guideline-inconsistent recommendations: older age (>70 years old), BMI ≥ 3 polyps, and having had ≥2 previous colonoscopies (Table 4).

When bowel preparation quality measured by the Aronchick scale, total BBPS score, or BBPS score for individual colon segments were added separately to the model, a fair bowel preparation on the Aronchick scale (8.89; 4.31–18.39), a BBPS = 5 (OR 15.26; 95% CI: 2.67–87.17), or having any colon segment with BBPS = 0-1 (5.62; 2.42–13.03) were associated with guideline-inconsistent recommendation. Also, noncompliance with split-dose preparation (OR = 2.4–2.9) and >12 hours or never between starting bowel preparation and the first clear bowel movement (OR = 2.6–3.1) remained significantly associated with guideline-inconsistent recommendation in all models (Table 4).

## 4. Discussion

Overuse of colonoscopy for CRC screening and colon polyp surveillance, defined as recommending repeat colonoscopy sooner than recommended by guidelines, has been described in multiple studies [1, 2, 4]. Minimizing this practice minimizes patients' exposure to the risks, inconvenience, and cost of colonoscopy. Furthermore, performing colonoscopy every 10 years after normal screening exams is necessary to maintain the cost-effectiveness of colonoscopy [3, 4]. In our study, the frequency of guideline-inconsistent recommendations was 13.6% and suboptimal bowel preparation was associated with guideline-inconsistent recommendations in adjusted multivariate logistic regression analysis. Specifically, fair bowel preparation by the Aronchick scale (OR = 8.89; 95% CI: 4.31–18.34) and BBPS = 5 (OR = 15.26; 95% CI: 2.67–87.17) were associated with recommendations to return early for colonoscopy. Our study also identified modifiable patient-related factors that were associated with guideline-inconsistent recommendations: noncompliance with split-dose preparation (OR = 2.89; 95% CI: 1.29–6.45) and requiring >12 hours to have the first clear bowel movement or never having a clear stool (OR = 3.13; 95% CI: 1.6–6.10). These findings, which have not been assessed in prior prospective studies after institution of split-dose bowel preparation, may guide potential interventions for quality improvement programs and future research.

Other patient-related and colonoscopy-related factors associated with guideline-inconsistent recommendations include patients >70, increasing BMI, ≥3 polyps, and ≥2 previous colonoscopies. These associations seem to be clinically intuitive from an endoscopist's perspective. Advancing age and increasing BMI are both associated with a higher likelihood of adenomas; endoscopists, therefore, may place these subjects in higher risk categories [20, 21]. Patients with ≥3 polyps found at colonoscopy may also elevate the endoscopist's index of suspicion for missed adenomas or future adenoma recurrence. The NCI Pooling Project found a linear increase in risk for advanced and nonadvanced neoplasia with each additional adenoma while the VA cooperative study 380 showed an increased risk of advanced neoplasia with the multiplicity of adenomas [22, 23]. While some or all of these factors might heighten endoscopists' suspicions or increase their level of concern, these concerns should not supersede evidence-based guideline recommendations in most patients. Conversely, endoscopists should remember that 100% adherence to these recommendations is not expected. The ACG/ASGE position statement on quality indicators for colonoscopy [4] targets 90% adherence, so endoscopists can individualize care for 10% of patients.

In our study, adherence was 86.4%. In order to improve adherence, interventions need to be identified and implemented. Split-dose noncompliance or requiring >12 hours to produce a clear bowel movement or never having a clear liquid stool during bowel preparation is potentially a modifiable factor. Therefore, future research may assess efficacy of having patients consume additional laxatives if they have not passed clear liquid stool within 12 hours of starting their bowel preparation. Multiple interventions improve patient compliance with bowel preparation including educational videos, phone calls, and education booklets [24–28], so quality improvement programs may consider implementing these interventions. Following implementation, QI programs could then track quality of bowel preparation and adherence to guideline recommendations.

Our study has significant strengths and limitations. Our findings are congruent with other retrospective studies demonstrating that suboptimal bowel preparation is associated with nonadherence to guideline recommendations [5–7, 29, 30]. It is a prospective study that utilized the BBPS, a validated bowel preparation quality measure for colonoscopy-oriented research, in addition to the commonly used Aronchick scale. Although Medicare claims database studies of overuse of colonoscopy can assess a much larger sample of patients, these studies may not be able to fully account for bowel preparation quality as a contributing factor [2, 31]. This study also was performed after institution of split-dose bowel preparation. However, this research was performed at an academic center, which may limit the generalizability of our findings for endoscopists, patients, and practice settings. Our sample size is relatively small, so this research need to be replicated in other settings in order to confirm or refute our findings.

In conclusion, gastroenterologists in this study were adherent with guidelines for timing of repeat colonoscopy in more than 85% of patients, but did not attain the recommended target of 90% adherence. Suboptimal bowel preparation is associated with guideline-inconsistent recommendations, and patient-modifiable factors were identified that may minimize this outcome while also improving quality of bowel preparation. Since this is a reportable quality indicator in PQRS and will probably be incorporated into CMS value-based index for reimbursement of colonoscopy, this issue is likely to garner closer scrutiny. Therefore, additional research about modifiable factors that are associated with guideline-inconsistent recommendations and research about interventions to minimize this outcome will be helpful to maximize the quality of CRC screening with colonoscopy.

## Figures and Tables

**Table 1:** Guideline recommendations (2006, 2008) utilized for follow-up colonoscopy intervals after screening colonoscopy in average-risk patient.

Colonoscopy finding	Follow-up interval (years)
No polyps	10
Any colonoscopy with poor/inadequate bowel prep	≤1
Small (<10 mm) hyperplastic polyps in rectum or sigmoid	10
1-2 small (<10 mm) tubular adenomas	5–10
3–10 tubular adenomas	3
>10 tubular adenomas	<3
One or more tubular adenomas ≥10 mm	3
One or villous adenomas	3
Adenoma with high-grade dysplasia	3
Poor/inadequate bowel preparation	≤1
Piecemeal resection of adenoma, if question of resection of completeness	≤1

**Table 2:** Study participant characteristics by whether physician recommendation was guideline compliant or not (*N* = 456).

Patient characteristic	Compliant (*n* = 394; 86%)	Noncompliant (*n* = 62; 14%)	Total (*N* = 456)	*p* value^a^
*Demographic characteristics*				
Age in years, mean (SD)	56.6 (6.6)	57.7 (8.2)	56.7 (6.8)	0.22
Male	196 (49.9)	33 (53.2)	229 (50.3)	0.62
White	340 (86.3)	54 (87.1)	394 (86.4)	0.86
Married	286 (72.6)	41 (66.1)	327 (71.7)	0.29
BMI^b^, kg/m^2^, mean (SD)	28.7 (5.7)	30.3 (6.0)	28.9 (5.7)	0.04
Full-time employment	232 (58.9)	31 (50.0)	263 (57.7)	0.19
On Medicaid or no insurance	16 (4.2)	3 (5.0)	19 (4.3)	0.76
≤75K annual income	153 (40.4)	29 (50.9)	182 (41.7)	0.13
*Health-related measures*				
Very good or excellent health	264 (67.9)	35 (56.5)	299 (66.3)	0.08
History of stroke	10 (2.6)	2 (3.2)	12 (2.7)	0.67
On prescription pain pills	23 (5.9)	7 (11.3)	30 (6.6)	0.16
On tricyclic antidepressant^c^	5 (1.3)	1 (1.6)	6 (1.3)	0.58
History of constipation	69 (17.8)	12 (19.4)	81 (18.0)	0.77

All values are *N* (% of split-dose compliant or % of split-dose noncompliant), unless otherwise specified. The total number of patients for each characteristic may not add to the total (*N* = 462) due to missing data. ^a^From testing differences in the distribution of the patient characteristics between patient compliant versus noncompliant to split-dose guideline, based on *t*-test for continuous variables and chi-square test or Fisher's exact test for categorical variables. No adjustments for multiple testing were done as the tests were not meant to be inferential, but to identify variables that are potentially associated with guideline-compliant physician recommendation. ^b^Body mass index ^c^on any of Tofranil, Elavil, Norpramin, Sinequan, and Pamelor.

**Table 3:** Distribution of bowel preparation-related variables by whether physician recommendation was guideline compliant or not (*N* = 456).

Bowel preparation characteristics	Compliant (*n* = 394)	Noncompliant (*n* = 62)	Total (*N* = 456)	*p* value^a^
BBPS total score				
BBPS ≥ 6	374 (94.9)	52 (83.9)	426 (93.4)	
BBPS = 5	3 (0.76)	7 (11.3)	10 (2.1)	
BBPS < 5	17 (4.3)	3 (4.8)	20 (4.3)	<0.001
BBPS segment score				
2 or greater for each segment	371 (94.2)	45 (72.6)	416 (91.2)	
0/1 in any segment	23 (5.8)	17 (27.4)	40 (8.8)	<0.001
Aronchick scale				
Excellent/good prep quality	333 (86.7)	29 (49.2)	362 (79.3)	
Fair prep quality	35 (9.1)	28 (47.5)	63 (13.8)	
Inadequate/poor prep quality	16 (4.2)	2 (3.4)	18 (3.9)	<0.001
Noncompliance to split-dose preparation	54 (13.7)	17 (27.4)	385 (84.4)	0.006
Number of polyps				
0	207 (52.5)	28 (45.2)	235 (51.5)	
1	95 (24.1)	8 (12.9)	103 (22.6)	
2	43 (10.9)	6 (9.7)	49 (10.8)	
3	19 (4.8)	8 (12.9)	27 (5.9)	
4	12 (3.1)	5 (8.1)	17 (3.7)	
≥5	18 (4.6)	7 (11.3)	25 (5.5)	0.004
Endoscopy suite				
Ambulatory surgery centers	184 (46.7)	24 (38.7)	208 (45.6)	
Academic hospital unit	210 (53.3)	38 (61.3)	248 (54.4)	0.24
First colonoscopy	250 (63.8)	36 (58.1)	286 (63.0)	0.39
Had ≥2 previous colonoscopies	29 (7.4)	14 (22.6)	43 (9.5)	<0.001
Followed laxative instructions exactly	264 (67.0)	37 (60.0)	301 (66.0)	0.26
Bowel prep type				
PEG	143 (36.5)	24 (38.7)	167 (36.8)	
MiraLAX®/Gatorade®	226 (57.7)	37 (59.7)	263 (57.9)	
Other^b^	23 (5.9)	1 (1.6)	24 (5.3)	0.38
Colonoscopy appointment was before 10:30 am.	193 (49.0)	22 (35.5)	215 (47.2)	0.05
>12 hours or never till clear BM	139 (35.6)	30 (49.2)	169 (37.5)	0.04

All values are *N* (% of split-dose compliant or % of split-dose noncompliant), unless otherwise specified. ^a^Please see footnote under Table 2. ^b^Includes sodium phosphate/Osmoprep, Half-Lytely, Moviprep, and 2-day prep.

**Table 4:** Adjusted odds ratios (95% confidence intervals) based on logistic regression models for guideline-compliant physician recommendation.

Patient characteristic	Model 1			Model 2 with BBPS Total score			Model 3 with BBPS segment score			Model 4 with Aronchick		
	AOR	95% Cl	*p* value	AOR	95% Cl	*p* value	AOR	95% Cl	*p* value	AOR	95% Cl	*p* value
Noncompliant to split-dose preparation	3.6	(1.7–7.7)	0.001	2.9	(1.3–6.5)	0.01	2.4	(1.1–5.4)	0.03	2.5	(1.1–5.9)	0.03
BBPS total												
≥6	—	—	—	1.0	Reference	—	—	—	—	—	—	—
=5	—	—	—	15.26	(2.7–87.2)	0.00	—	—	—	—	—	—
<5	—	—	—	1.35	(0.3–5.8)	0.68	—	—	—	—	—	—
BBPS segment												
≥2	—	—	—	—	—	—	1.0	Reference	—	—	—	—
0/1	—	—	—	—	—	—	5.62	(2.4–13.0)	0.00	—	—	—
Aronchick												
≥Good	—	—	—	—	—	—	—	—	—	1.0	Reference	—
Fair	—	—	—	—	—	—	—	—	—	8.9	(4.3–18.3)	0.00
Poor	—	—	—	—	—	—	—	—	—	1.4	(0.3–7.7)	0.70
Age > 70 years old	3.6	(1.2–10.6)	0.02	3.4	(1.3–11.2)	0.02	3.7	(1.2–11.6)	0.02	4.9	(1.4–16.6)	0.01
Male	1.2	(0.6–2.2)	0.59	1.1	(0.6–2.2)	0.58	1.2	(0.6–2.2)	0.6	1.2	(0.6–2.3)	0.59
White	1.4	(0.6–3.5)	0.48	1.5	(0.6–3.7)	0.41	1.8	(0.7–4.9)	0.24	1.4	(0.5–3.8)	0.53
Endoscopy at hospital a	1.2	(0.7–2.2)	0.54	1.3	(0.7–2.5)	0.37	1.2	(0.7–2.3)	0.51	1.3	(0.6–2.4)	0.51
Number of polyps: 0	1.0	Reference		1.0	Reference		1.0	Reference		1.0		
1	0.7	(0.3–1.6)	0.35	0.7	(0.3–1.6)	0.36	0.7	(0.3–1.6)	0.34	0.7	(0.3–2.0)	0.54
2	1.0	(0.4–2.7)	0.98	1.0	(0.3–2.7)	0.94	0.9	(0.3–2.7)	0.87	1.2	(0.4–3.6)	0.77
3	3.0	(1.4–6.3)	0.005	3.2	(1.5-6.9)	0.003	3.3	(1.5–7.0)	0.00	4.4	(2.0–10)	0.00
Had ≥2 previous colonoscopies	3.7	(1.6–8.7)	0.003	3.3	(1.4–8.0)	0.007	3.0	(1.2–7.3)	0.02	2.5	(0.9–6.5)	0.07
>12 hours or never till clear BM	3.4	(1.8–6.6)	<0.001	3.1	(1.6–6.1)	0.002	2.8	(1.4-5.4)	0.00	2.6	(1.3–5.4)	0.01
Body mass index	1.1	(1.0-1.1)	0.03	1.1	(1.0-1.1)	0.04	1.1	(1.0-1.1)	0.02	1.1	(1.0-1.1)	0.10

AOR: adjusted odds ratio; CI: confidence interval; BM: bowel movement. a versus ambulatory surgery centers.

## Data Availability

The data used to support the findings of this study are included within the article.

## Abbreviations

CRC: Colorectal cancer screening
GI: Gastrointestinal.
